# Upstroke Time Per Cardiac Cycle as A Novel Parameter for Mortality Prediction in Patients with Acute Myocardial Infarction

**DOI:** 10.3390/jcm9040904

**Published:** 2020-03-25

**Authors:** Po-Chao Hsu, Wen-Hsien Lee, Wei-Chung Tsai, Ying-Chih Chen, Nai-Yu Chi, Ching-Tang Chang, Chun-Yuan Chu, Tsung-Hsien Lin, Chee-Siong Lee, Wen-Ter Lai, Sheng-Hsiung Sheu, Ho-Ming Su

**Affiliations:** 1 Division of Cardiology, Department of Internal Medicine, Kaohsiung Medical University Hospital, Kaohsiung 807, Taiwan; pochao.hsu@gmail.com (P.-C.H.); cooky-kmu@yahoo.com.tw (W.-H.L.); azygo91@gmail.com (W.-C.T.); 990329kmuh@gmail.com (Y.-C.C.); marchchi@gmail.com (N.-Y.C.); lcsphk@ms18.hinet.net (C.-S.L.); wtlai@kmu.edu.tw (W.-T.L.); sheush73@gmail.com (S.-H.S.); 2 Faculty of Medicine, College of Medicine, Kaohsiung Medical University, Kaohsiung 807, Taiwan; 3 Department of Internal Medicine, Kaohsiung Municipal Siaogang Hospital, Kaohsiung 812, Taiwan; 4 Research Center for Environmental Medicine, Kaohsiung Medical University, Kaohsiung 807, Taiwan

**Keywords:** acute myocardial infarction, cardiovascular, mortality, upstroke time per cardiac cycle

## Abstract

Background: Acute myocardial infarction (AMI) is one of the leading causes of death in the world. How to simply predict mortality for AMI patients is important because the appropriate treatment should be done for the patients with higher risk. Recently, a novel parameter of upstroke time per cardiac cycle (UTCC) in lower extremities was reported to be a good predictor of peripheral artery disease and mortality in elderly. However, there was no literature discussing the usefulness of UTCC for prediction of cardiovascular (CV) and overall mortality in AMI patients. Methods: 184 AMI patients admitted to the cardiac care unit were enrolled. Ankle-brachial index (ABI) and UTCC were measured by an ABI-form device in the same day of admission. Results: The median follow-up to mortality was 71 months. There were 36 CV and 124 overall mortality. Higher UTCC was associated with increased CV and overall mortality after multivariable analysis (*P* = 0.033 and *P* < 0.001, respectively). However, ABI was only associated with CV mortality and overall mortality in the univariable analysis but became insignificant after the multivariable analysis. In addition, after adding UTCC into a basic model including important clinical parameters, left ventricular ejection fraction, Charlson comorbidity index, and ABI, we found the basic model + UTCC had a better predictive value for overall mortality than the basic model itself (*P* < 0.001). Conclusions: Our study is the first one to evaluate the usefulness of UTCC in AMI patients for prediction of long-term mortality. Our study showed UTCC was an independent predictor of long-term CV and overall mortality and had an additive predictive value for overall mortality beyond conventional parameters. Therefore, screening AMI patients by UTCC might help physicians to identify the high-risk group with increased mortality.

## 1. Introduction

Acute myocardial infarction (AMI) is an important public health issue in the world [1,2]. AMI includes ST segment elevation myocardial infarction (STEMI) and non-ST elevation myocardial infarction (NSTEMI). In Taiwan, the incidence of NSTEMI continues to increase in recent years and the cases of NSTEMI outnumbered STEMI [3]. Electrocardiogram (ECG) and cardiac enzymes are important tools to help physicians make differential diagnosis for STEMI and NSTEMI. ECG findings in STEMI reveal ST segment elevation or newly-onset left bundle branch block. However, ECG findings in NSTEMI show absence of ST segment elevation. Although the cardiovascular (CV) outcome of AMI has been improved by the advance of percutaneous coronary intervention and evidence-based medication, patients with AMI still have a relative high CV morbidity and mortality. 

Upstroke time (UT) measured from the beginning of the systolic rise to the peak of the pulse wave in the lower extremity was reported to be a good predictor of peripheral artery disease (PAD) [4,5,6], and UT per cardiac cycle (UTCC) was shown to be a diagnostic tool of PAD and even a predictor of mortality in elderly [7]. UTCC can be easily measured by the ankle-brachial index (ABI)-form device in few minutes. Because PAD is one of the major atherosclerotic disease and shares similar risk factors with cerebrovascular disease and coronary artery disease including AMI, and patients with one vascular bed disease often have coexistent diseases in other vascular beds, the novel parameter of UTCC may also play an important role in prediction of long-term CV outcome for AMI patients. However, there was no literature discussing whether UTCC can predict long-term CV and overall mortality in AMI patients. Therefore, we conducted this study to evaluate the issue.

## 2. Materials and Methods

### 2.1. Study Population and Design

This observational cohort study consecutively included AMI (STEMI and NSTEMI) patients admitted to our cardiac care unit from November 2003 to September 2004. Patients with age ≥ 20 years old were eligible for inclusion. Patients with atrial fibrillation, limb amputation, and missing data of four-limb blood pressures, ABI, and UTCC were excluded. Finally, 184 AMI patients were included in this study. CV and overall mortality data were collected up to December 2018. Mortality data were obtained from the Collaboration Center of Health Information Application (CCHIA), Ministry of Health and Welfare, Executive Yuan, Taiwan.

### 2.2. Ethics Statement

The study protocol was approved by the institutional review board (IRB) committee of our hospital. Informed consents were obtained in written form from patients during the first day of admission and all clinical investigation was conducted according to the principles expressed in the Declaration of Helsinki. 

### 2.3. Assessment of ABI, UTCC, and Four Limb Blood Pressures by ABI-form Device 

The ABI, UTCC, and four limb blood pressures were measured by using an ABI-form device (VP1000; Colin Co. Ltd., Komaki, Japan), which automatically and simultaneously measures blood pressures in both arms and ankles using an oscillometric method [8,9]. ABI of each leg was calculated by the ratio of the ankle over the higher brachial systolic blood pressure. After obtaining bilateral ABIs, the lower one was selected for later analysis. Upstroke time was reported by the device as the pulse foot-to-peak transit time. UTCC was calculated as the upstroke time divided by cardiac cycle [7,10]. We calculated the UTCC of the bilateral foot separately and the higher one was selected for analysis. The ABI-form device measurement was done once in each patient and was performed within 24 h of admission to the cardiac care unit.

### 2.4. Collection of Demographic and Medical Data

Demographic and medical data including age, gender, and comorbid conditions such as dyslipidemia, diabetes, hypertension, and Charlson comorbidity index (CCI) were obtained from medical records. 

### 2.5. Statistical Analysis

All statistical analyses were performed with SPSS 22.0 software (SPSS, Chicago, IL, USA). Data were expressed as mean ± standard deviation, percentage, or median (25th–75th percentile) for follow-up period. Continuous and categorical variables between groups were compared by independent samples t-test and Chi-square test, respectively. The significant variables in the univariable analysis were selected for multivariable analysis. Time to the CV and overall mortality and covariates of risk factors were adjusted using a Cox proportional hazards model. The incremental value of UTCC over basic model including age, gender, hypertension, heart rate, body mass index, left ventricular ejection fraction (LVEF), CCI, and ABI in prediction of overall and CV mortality was studied by calculating the improvement in global Chi-square value. The Kaplan–Meier survival plot was calculated from baseline to time of mortality events. All tests were 2-sided and *P* value less than 0.05 was considered statistically significant.

## 3. Results

Among the 184 subjects, mean age was 65.6 ± 13.6 years. There were 37 and 147 patients with STEMI and NSTEMI, respectively. The median follow-up to mortality was 71 months (25th–75th percentile: 8–174 months). There were 36 and 124 patients documented as CV and overall mortality, respectively. 

Table 1 compares the clinical characteristics between patients with UTCC below and above the median (19.2%). Compared with patients with UTCC below the median, patients with UTCC above the median had older age, female gender, higher prevalence of hypertension and PAD, higher heart rate, higher CCI, lower ABI (1.04 ± 0.11 versus 0.86 ± 0.22, *P* < 0.001), and higher UTCC (16.1 ± 2.07 versus 25.9 ± 7.93, *P* < 0.001). There was no significant difference in prevalence of dyslipidemia, diabetes mellitus, and STEMI, LVEF, and body mass index between the two groups.

Table 2 shows the predictors of CV mortality using the Cox proportional hazards model in the univariable and multivariable analysis. In the univariable analysis, older age, female gender, higher prevalence of hypertension, higher heart rate, lower LVEF, higher CCI, lower ABI, and higher UTCC were significant predictors of CV mortality. After multivariable analysis, only the prevalence of hypertension and UTCC (hazard ratio (HR) = 1.844; 95% confidence interval (CI): 1.018–3.342; *P* = 0.043) were still significantly associated with CV mortality. The ABI value became insignificant after multivariable analysis (*P* = 0.407).

Table 3 shows the predictors of overall mortality using Cox proportional hazards model in the univariable and multivariable analysis. In the univariable analysis, older age, female gender, prevalence of hypertension, higher heart rate, lower body mass index, lower LVEF, higher CCI, lower ABI, and higher UTCC were significant predictors of overall mortality. After multivariable analysis, only age and UTCC (HR = 1.849; 95% CI: 1.367–2.501; *P* < 0.001) were still significantly associated with overall mortality. Similarly, the ABI value became insignificant after multivariable analysis (*P* = 0.493). 

**Figure 1:**
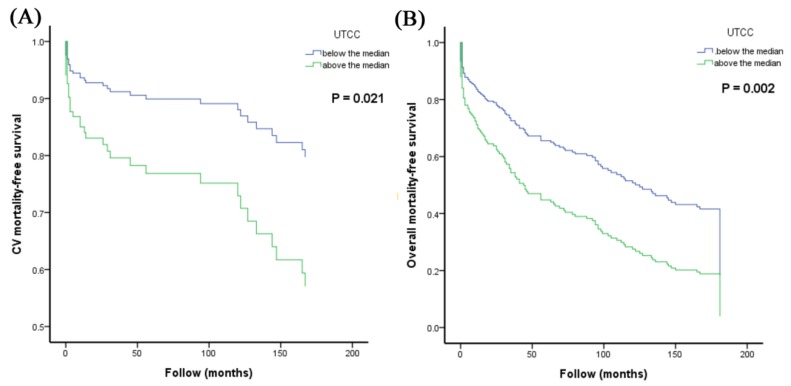
Adjusted Kaplan–Meier curves of UTCC above the median versus below the median for CV (**A**) and overall mortality-free survival (**B**). Abbreviation: CV, cardiovascular; UTCC, upstroke time per cardiac cycle.

Figure 1 illustrates the adjusted Kaplan–Meier curves of UTCC above the median versus below the median for CV (Figure 1A) and overall mortality-free survival (Figure 1B).

**Figure 2:**
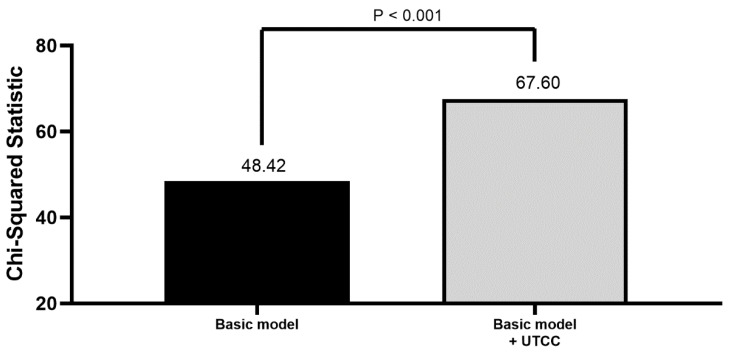
The additive effect of UTCC on overall mortality prediction by calculating the improvement in global Chi-square value. Basic model included age, gender, hypertension, heart rate, body mass index, left ventricular ejection fraction, Charlson comorbidity index, and ankle-brachial index. Abbreviation: UTCC, upstroke time per cardiac cycle.

Figure 2 shows the additive effect of UTCC on overall mortality prediction by calculating the improvement in global Chi-square value. The variables in the basic model included age, gender, hypertension, heart rate, body mass index, LVEF, CCI, and ABI. After adding UTCC into the basic model, we found basic model + UTCC had a better predictive value for overall mortality than basic model itself (*P* < 0.001). 

In addition, we also evaluated the association between ABI and UTCC and found that UTCC was significantly correlated with ABI (r = −0.558, *P* < 0.001).

## 4. Discussion

Our study aimed to evaluate the usefulness of UTCC on the prediction of CV and overall mortality in AMI patients. There were several major findings in the present study. First, higher UTCC was associated with increased CV and overall mortality in univariable and multivariable analysis. Second, although ABI was associated with increased CV and overall mortality in the univariable analysis, it became insignificant after multivariable analysis. Third, UTCC had an additive predictive value beyond conventional parameters for overall mortality prediction in AMI patients.

AMI is a CV emergency and an important public health issue despite the utilization of percutaneous coronary intervention and evidence-based medications [1]. Because AMI is complicated with high morbidity and mortality in the world, finding an easy and simple tool for long-term mortality prediction for AMI patients became so important. 

A clinical device, ABI-form (VP 1000; Colin Co Ltd., Komaki, Japan), has been developed to simultaneously and automatically measure blood pressures in four limbs and record pulse waves of the brachial and posterior tibial arteries using an automated oscillometric method. Using this device, we can not only obtain the values of four limb blood pressures and ABI, but also UT by analyzing the signals of phonocardiogram, electrocardiogram, and brachial pressure volume waveform. ABI is frequently used as a tool for diagnosis of PAD, and it is considered as a powerful predictor of CV outcome [11,12,13,14,15]. UT was previously reported to have an important role for diagnosis of PAD [4,5], and it also had a significant correlation with ABI in the literature [7,10]. Recently, Sheng CS et al. reported that UTCC was equivalent to ABI for diagnosis of PAD and could be used as a novel parameter for mortality prediction in the Chinese elderly [7]. In addition, Yu S et al. also showed that UTCC was significantly associated with vascular and renal damage in the Chinese elderly while comparing with ABI [10]. In our study, patients with UTCC above the median had a higher prevalence of PAD than patients with UTCC below the median (45.7% vs. 6.5%). In addition, we also found UTCC was significantly correlated with ABI (r = −0.558, *P* < 0.001), which was similar to previous studies [7,10]. According to the literature, patients with PAD had a higher mortality than patients with AMI [16,17]. Sartipy F et al. also reported that 10 years all-cause mortality was 56% for asymptomatic PAD, 63% for intermittent claudication, and 75% for severe limb ischemia [17]. This is one possible rationale why patients with a higher UTCC may have a higher mortality. Furthermore, Manfredini F et al. also found rehabilitative exercise reduced the peripheral revascularization and deaths in patients with severe PAD, especially for those with significant ABI increase [18]. The above studies might suggest patients with PAD had a high mortality rate and treating PAD effectively could reduce mortality. Therefore, our study tried to investigate whether UTCC was an excellent parameter for prediction of long-term CV and overall mortality in AMI patients. In our study, increased UTCC was significantly associated with increased CV and overall mortality in the univariable and multivariable analysis. However, ABI became insignificant after multivariable analysis. In addition, our study also showed that UTCC had an additive predictive value beyond conventional parameters for long-term mortality prediction by calculating the improvement in global Chi-square value. Therefore, UTCC as a novel parameter might play a more important role than ABI for prediction of CV and overall mortality in AMI patients. 

### Study Limitations

There were some limitations to this study. First, the sample size of our study was not very large, but the follow-up period was very long, up to 181 months. Second, we did not adjust medications in the multivariable analysis because of incomplete data. In our hospital, the patient chart was not available if he or she did not visit our hospital again more than 10 years. 

## 5. Conclusions

Our study is the first one to evaluate the usefulness of UTCC in AMI patients for prediction of long-term CV and overall mortality. Our study showed UTCC could be used as a novel parameter to predict long-term CV and overall mortality. In addition, it also had an additive predictive value for overall mortality beyond conventional parameters in AMI patients. Therefore, screening AMI patients by UTCC might help physicians to identify the high-risk group with increased mortality. 

## Figures and Tables

**Table 1:** Baseline characteristics of the study population classified by upstroke time per cardiac cycle (UTCC) below and above the median (19.2%).

Baseline Characteristics	UTCC below the Median	UTCC above the Median	*P* value
Number	92	92	
Age (years)	60 ± 13	71 ± 12	<0.001
Male gender (%)	82.6%	62.0%	0.003
Dyslipidemia (%)	26.1%	37.0%	0.153
Diabetes mellitus (%)	23.9%	30.4%	0.407
Hypertension (%)	31.5%	54.3%	0.003
STEMI (%)	19.6%	20.7%	1.000
NSTEMI (%)	80.4%	79.3%	1.000
PAD (%)	6.5%	45.7%	<0.001
Heart rate (beat/min)	70.3 ± 12.3	87.4 ± 17.3	<0.001
LVEF	60.9 ± 15.2	55.8 ± 15.6	0.066
CCI	2.38 ± 1.64	3.95 ± 1.79	<0.001
Body mass index (kg/m^2^)	24.7 ± 3.6	24.0 ± 4.0	0.231
Ankle brachial index	1.04 ± 0.11	0.86 ± 0.22	<0.001
UTCC (%)	16.1 ± 2.07	25.9 ± 7.93	<0.001

Abbreviations: CCI, Charlson comorbidity index; LVEF, left ventricular ejection fraction; NSTEMI, non-ST elevation myocardial infarction; PAD, peripheral artery disease; STEMI, ST-segment elevation myocardial infarction; UTCC, upstroke time per cardiac cycle.

**Table 2:** Predictors of cardiovascular mortality using the Cox proportional hazards model by univariable and multivariable analysis.

Parameter	Univariable Analysis		Multivariable Analysis	
	HR (95% CI)	*P*	HR (95% CI)	*P*
Age (Per 1 year)	1.049(1.020–1.079)	< 0.001	-	0.801
Gender (Male vs. Female)	0.481(0.245–0.944)	0.033	-	0.406
Diabetes mellitus (Yes vs. No)	1.154(0.556–2.295)	0.700	-	-
Hypertension (Yes vs. No)	2.464(1.258–4.828)	0.009	3.363(1.163-9.731)	0.025
Dyslipidemia (Yes vs. No)	0.972(0.478–1.975)	0.937	-	-
STEMI (Yes vs. No)	0.947(0.415–2.164)	0.898	-	-
Heart rate (Per beat/min)	1.020(1.001–1.038)	0.036	-	0.707
Body mass index (Per 1kg/m^2^)	0.950(0.864–1.043)	0.282	-	-
LVEF (Per 1%)	0.973(0.947–1.000)	0.048	-	0.355
CCI	1.400(1.198–1.637)	< 0.001	-	0.943
Ankle brachial index (Per 1SD)	0.656(0.489–0.881)	0.005	-	0.407
UTCC (Per 1SD)	1.627(1.231–2.151)	0.001	1.844(1.018–3.342)	0.043

HR: hazard ratio; CI: confidence interval; SD: standard deviation; other abbreviations as in Table 1. SD for ankle brachial index was 0.208; SD for UTCC was 7.597.

**Table 3:** Predictors of overall mortality using Cox proportional hazards model by univariable and multivariable analysis.

Parameter	Univariable Analysis		Multivariable Analysis	
	HR (95% CI)	*P*	HR (95% CI)	*P*
Age (Per 1 year)	1.070(1.053–1.087)	<0.001	1.050(1.028–1.073)	<0.001
Gender (Male vs. Female)	0.606(0.416–0.883)	0.009	-	0.339
Diabetes mellitus (Yes vs. No)	1.173(0.793–1.736)	0.424	-	-
Hypertension (Yes vs. No)	1.570(1.103–2.235)	0.012	-	0.703
Dyslipidemia (Yes vs. No)	1.072(0.735–1.564)	0.717	-	-
STEMI (Yes vs. No)	0.922(0.590–1.440)	0.721	-	-
Heart rate (Per 1beat/min)	1.019(1.009–1.029)	<0.001	-	0.956
Body mass index (Per 1kg/m^2^)	0.902(0.856–0.950)	<0.001	-	0.149
LVEF (Per 1%)	0.983(0.970–0.997)	0.014	-	0.150
CCI	1.438(1.322–1.564)	< 0.001	-	0.506
Ankle brachial index (Per 1SD)	0.790(0.727–0.858)	< 0.001	-	0.493
UTCC (Per 1SD)	1.084(1.063–1.105)	< 0.001	1.849(1.367–2.501)	<0.001

HR: hazard ratio; CI: confidence interval; SD: standard deviation; other abbreviations as in Table 1. SD for ankle brachial index was 0.208; SD for UTCC was 7.597.
